# Mechanotransduction for Muscle Protein Synthesis via Mechanically Activated Ion Channels

**DOI:** 10.3390/life13020341

**Published:** 2023-01-27

**Authors:** Timur M. Mirzoev

**Affiliations:** Myology Laboratory, Institute of Biomedical Problems RAS, 123007 Moscow, Russia; tmirzoev@yandex.ru

**Keywords:** mechanotransduction, mechanical stimulation, mechanically-activated channels, stretch-activated channels, protein synthesis, anabolic signaling, skeletal muscle

## Abstract

Cell mechanotransduction, the ability to detect physical forces and convert them into a series of biochemical events, is important for a wide range of physiological processes. Cells express an array of mechanosensors transducing physical forces into intracellular signaling cascades, including ion channels. Ion channels that can be directly activated by mechanical cues are known as mechanically activated (MA), or stretch-activated (SA), channels. In response to repeated exposures to mechanical stimulation in the form of resistance training, enhanced protein synthesis and fiber hypertrophy are elicited in skeletal muscle, whereas a lack of mechanical stimuli due to inactivity/mechanical unloading leads to reduced muscle protein synthesis and fiber atrophy. To date, the role of MA channels in the transduction of mechanical load to intracellular signaling pathways regulating muscle protein synthesis is poorly described. This review article will discuss MA channels in striated muscle, their regulation, and putative roles in the anabolic processes in muscle cells/fibers in response to mechanical stimuli.

## 1. Introduction

The ability to detect physical forces and convert them into a series of biochemical events (mechanotransduction) is particularly relevant to muscle cells/fibers that play a fundamentally mechanical role. Accordingly, skeletal muscle fibers are equipped with mechanosensory structures that are directly involved in the sensing of various mechanical forces and triggering various intracellular signaling events that control metabolic processes, including protein synthesis. An important role in the regulation of skeletal muscle mass in response to mechanical loading or unloading belongs to protein synthesis. The maintenance of skeletal muscle mass is vital for overall health since skeletal muscle is the largest organ by mass that contributes significantly to the prevention and cure of various diseases and problems linked to the quality of life [1,2]. Mechanical tension represents one of the most widely recognized factors that directly determines skeletal muscle mass in mammals. The first data that muscle cells or fibers are able to perceive the mechanical loading in nerve- and hormone-independent fashion came from in vitro and in vivo studies conducted back in the 20th century. In particular, Vandenburgh and Kaufman showed that static stretch can induce a significant increase in protein synthesis rates in chick skeletal muscle cells [3]. A similar phenomenon was observed in cardiac cells plated on a silicone sheet [4]. Sadoshima et al. (1992) characterized the stretch-induced adaptation of cultured neonatal cardiac cells grown in a serum-free medium. Using an in vitro model of load-induced cardiac hypertrophy, the authors demonstrated that non-injured cell-stretching leads to an increase in protein synthesis, assessed by [^3^H] phenylalanine incorporation, and subsequent hypertrophy of cardiac cells [5]. In the 1960s, Albert Goldberg for the first time demonstrated that pituitary growth hormone is dispensable for compensatory hypertrophy (due to mechanical overloading) of rat soleus muscle [6]. Moreover, Goldberg revealed that mechanically overloaded muscles show increased rates of protein synthesis compared to control muscles, and muscle mass gain is proportional to the increase in leucine—^14^C incorporation [7]. These results suggested that skeletal muscle likely possesses intrinsic sensors to sense and convert mechanical stimuli into an intracellular anabolic response. Conversely, a lack of mechanical tension under conditions such as microgravity, immobilization, and limb suspension leads to a substantial decrease in muscle protein synthesis rates, thereby contributing to the loss of muscle mass [8,9,10,11,12]. Thus, overloading/unloading-induced alterations in muscle protein synthesis play a fundamental role in the mechanical regulation of skeletal muscle mass.

Muscle cells/fibers express an array of mechanosensors that can be roughly divided into two groups: (1) sarcolemmal mechanosensors (stretch-activated ion channels, focal adhesion complexes associated with integrins and components of the dystrophin-glycoprotein complex), and (2) sarcomeric mechanosensors (several domains of a giant titin protein) [13,14,15,16]. It is also worth noting the concept of Donald Ingber, according to which the structure of any cell is organized according to the principle of tensegrity (tensional integrity) [17,18]. The tensegrity model explains how the cell can perceive an external mechanical signal applied locally to integrins/focal adhesions and simultaneously transmit it through the cytoskeleton to different parts of the cell (nucleus, mitochondria, etc.) [17,18]. Mechanically activated (MA) or stretch-activated (SA) ion channels constitute primary sarcolemmal sensors of mechanical forces. However, precise mechanisms of force transduction via these channels and their physiological role in the regulation of anabolic processes in striated muscle remain largely unknown. This review will discuss principal models of MA channel gating as well as recent developments and unresolved issues regarding a putative role of MA channels in the regulation of anabolic processes in skeletal muscle cells/fibers in response to mechanical stimuli. A better understanding of the physiological functions of MA channels in striated muscles will likely provide novel therapeutic strategies for the treatment of muscle atrophy [19], Duchenne muscular dystrophy (DMD) [20], heart failure, and cardiac hypertrophy [21].

## 2. MA Channels in Skeletal Muscle and Proposed Models of Their Activation

In skeletal muscle tissue, MA channels were first found by Guharay and Sachs (1984) by detection of SA single ion channel currents in the membrane of tissue-cultured chick pectoral muscle [22]. Later, Franco and Lansman (1990) revealed calcium (Ca^2+^) entry through mechano-transducing ion channels in mdx myotubes (i.e., myotubes lacking dystrophin) [23] and in developing muscle cells from a mouse cell line [24]. MA channels were also identified in isolated skeletal muscle fibers (murine flexor digitorum brevis muscle), and notably, single MA channels in isolated fibers had properties indistinguishable from those of muscle cells grown in tissue culture [25]. It was also established that, apart from Ca^2+^, MA channels from mouse muscle cells and cardiac myocytes are permeable to Li^+^, Na^+^, and K^+^ [24,26].

One of the most widely used inhibitors of MA ion channels is gadolinium (Gd^3+^) [24]. It has been demonstrated that Gd^3+^ can bind to charged phospholipids with high affinity, thereby producing strong electrostatic effects on the surface of the lipid bilayer [27]. This data suggested that gadolinium may exert its inhibitory effect on mechanically-activated channels by acting on membrane lipids rather than by binding to the channel pore. However, using mdx myotubes, Franco et al. (1991) suggested that Gd^3+^ can inhibit MA channels by plugging the open channels, thereby preventing ion flow [28]. It is important to note that Gd^3+^ is relatively nonspecific, as Gd^3+^ is able to block L-type calcium channels, store-dependent calcium channels, and Cl^−^ channels, although generally with lower potency [20]. In addition, MA ion channels can be inhibited by aminoglycoside antibiotics. In skeletal muscle, aminoglycosides (such as neomycin and streptomycin) block both the L-type channel and MA channel [29]. Blocking of MA channels with aminoglycosides involves a partial occlusion of the channel pore at high concentrations. At the same time, *Grammostola spatulata* mechano-toxin #4 (GsMTx4), a peptide isolated from the venom of the Chilean rose tarantula spider, is currently considered the most potent and specific inhibitor of cationic MA ion channels [30,31,32].

Considering MA ion channels, questions arise about the molecular identity of these channels. Initially, it was claimed that transient receptor potential (TRP) canonical channels (such as TRPC1 and TRPC6) might encode the mammalian MA channels [33,34]. In 2005, Maroto and colleagues found in frog oocytes that TRPC1 protein is a component of MA channels, the activity of which is regulated by the tension of the lipid bilayer [33]. These authors showed that “heterologous expression of the human TRPC1 resulted in a >1000% increase in mechanosensitive cation channel patch density, whereas the injection of a TRPC1-specific antisense RNA abolished endogenous mechanosensitive cation channel activity” [33]. Furthermore, transfection of human TRPC1 into Chinese hamster ovary (CHO) cells also significantly increased SA channel expression [33]. TRPC1 is widely expressed and present in both cardiac and skeletal muscle [35]. It was also demonstrated that TRPC1 represents a non-selective store-operated ion channel involved in the Ca^2+^ entry due to the depletion of Ca^2+^ in the sarcoplasmic reticulum [35]. TRPC6 is another Ca^2+^-permeable cation channel that is known to be expressed in striated muscles [35,36]. Hofmann et al. (1999) showed that TRPC6 could be directly activated by diacylglycerol (DAG) in response to phosphatidylinositol 4,5-bisphosphate (PIP2) hydrolysis by stimulation of different receptors in the plasmalemma, hence named a “receptor-operated channel” (ROC) [37]. It has also been demonstrated that TRPC6 is able to sense membrane stretch induced by mechanical and osmotic pressure. In addition, TRPC6 may be inhibited by GsMTx-4, a well-known blocker of cation MA ion channels [35]. Membrane tension has been proposed to directly gate TRPC6 [35]. DAG (TRPC6 activator) and GsMTx-4 (TRPC6 blocker) may differentially affect the mechanical deformation of the plasmalemma, thereby modulating TRPC6 activity [35].

At the same time, some literature data do not support the notion of the direct mechanosensitivity of TRP channels [38,39,40]. Evidence suggests that TRPC1 and TRPC6 are not genuine MA channels and that plasmalemma stretching does not primarily gate these channels [23,40,41]. It is not excluded that stretch sensitivity of these channels is indirect and may require an additional component in the membrane, for instance, angiotensin II receptor type 1 (AT1) [35].

In 2010, Patapoutian’s research group identified the Piezo family of proteins (Piezo1 and Piezo2) that function as bona fide MA ion channels [41]. In 2016, the same scientific group reliably showed that the stimulus for the activation of the ion channel formed by the Piezo1 protein is a direct deformation of the lipid bilayer in the membrane [42]. It was found that Piezo channels play a key role in the sensation of touch, as well as proprioception [43]. It is encouraging to note that the discovery of the Piezo channels and their physiological role in the body has led to the 2021 Nobel Prize in Physiology or Medicine. It was also found that the synthetic molecule Yoda1 can serve as a specific agonist of Piezo1 channels [44]. The functions of Piezo1 in skeletal muscles remain poorly understood. Currently, few papers concerning the role of Piezo1 in skeletal muscle cells have been published. A group of researchers from Japan have shown (in C2C12 cells) an important role of Piezo1 activation for morphogenesis during the formation of myotubes (myoblast fusion) [45]. A recent study characterized the cellular localization of Piezo1 in primary muscle satellite cells, myotubes, and muscle fibers, as well as analyzed the effect of Piezo1 activation at the key stages of myogenesis [46]. In skeletal muscle progenitor cells, the use of Piezo1 agonist (Yoda1) significantly stimulated cell differentiation and fusion, rather than the proliferation of satellite cells [46]. In cultured muscle myotubes, Sciancalepore et al. (2022) demonstrated the role of Piezo1 in myokine (interleukin 6, IL-6) release [47]. However, skeletal muscle immobilization was recently found to be associated with Piezo1 downregulation and subsequent upregulation of IL-6 via transcription factor Krüppel-like factor-15 [19]. The discrepancy between the reported studies deserves further investigation.

**Figure 1:**
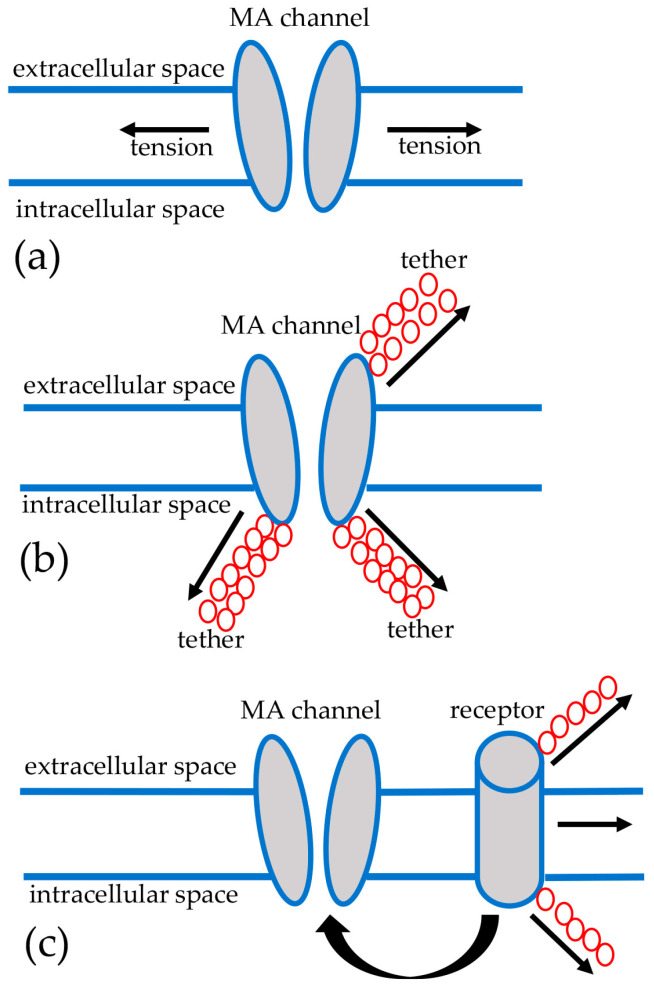
Proposed models of MA ion channels activation. (**a**) Mechanical tension of the membrane is transmitted to a MA channel via the surrounding lipid bilayer (force-from-lipids model). (**b**) Tensions may be transferred from the ECM or cytoskeletal proteins to the MA channel via tethers/gating springs (force-from-filament model). (**c**) An indirect model of MA channels gating is based on the existence of some other sarcolemmal mechanosensors/receptors that might be influenced by the ECM and/or cytoskeleton and are able to release second messengers impacting the opening of MA channels.

According to current concepts, the activation of MA channels in response to mechanical stress can be carried out by (1) interacting with lipids surrounding the channel (force-from-lipids model), (2) interacting with proteins of the cytoskeleton and/or extracellular matrix (ECM) via tethers/gating springs (force-from-filament model) and (3) interacting with another primary mechanosensor (for example, angiotensin II receptor type 1 (AT1)), which may also be linked to the ECM or cytoskeleton and could involve the release of a second messenger (such as diacylglycerol) leading to the activation of a MA ion channel (indirect gating model) [35,48,49]. All three models of MA ion channel activation are shown in Figure 1.

## 3. Interaction of Cytoskeletal and Scaffolding Proteins with MA Channels: Cholesterol-Dependent Regulation of MA Channels

The activity of MA channels largely depends on the mechanical properties of the lipid bilayer [35]. MA channel activity is greatly impacted by lipid composition in the membrane and interaction with cytoskeletal proteins/extracellular matrix [35]. For example, destabilization of the cortical actin cytoskeleton in dystrophic myotubes results in changes in membrane tension and an increase in SA channel activity [23,50]. Cytoskeleton disruption is considered to enlarge the radius of plasmalemma curvature, thereby increasing tension in the lipid bilayer for a given pressure stimulus resulting in an increased MA channel activity [35]. Under conditions of DMD, the lack of dystrophin, a subsarcolemmal protein linking the actin cytoskeleton to the ECM, can significantly influence MA channel function. Vandebrouck et al. (2007) demonstrated in skeletal muscles from mdx mice that TRPC1, the content of which is increased under this condition, can interact with dystrophin and α1-syntrophin [51]. Increased Ca^2+^ entry through TRPC1 was proposed to contribute to cell damage in the skeletal muscles of mdx mice [52]. Importantly, the pharmacological blockade of MA channels with GsMTx4 is able to protect mdx mice-derived muscle fibers from stretching-induced cell damage and reduce intracellular calcium [53].

The literature suggests that actin cytoskeleton (network of stress-fibers) rearrangement can exert a significant impact on the activity of MA ion channels. However, the available data on the effect of the actin cytoskeleton on the activity of MA channels are contradictory. On the one hand, it has been shown that depolymerization/disruption of the actin cytoskeleton can contribute to an increase in the ion flow through MA ion channels [54,55,56]. On the other hand, there is evidence that depolymerization of actin stress-fibers contributes to a decrease in the conductivity (and activity) of mechanically activated ion channels [57,58,59,60]. One group of researchers also showed that the polymerization of actin and the formation of stress fibers in response to the treatment of C2C12 myoblasts with sphingosine 1-phosphate increases the ion current through MA ion channels [61]. Later, it was shown that the key role in activating these channels is played not so much by the formation of the actin stress-fibers per se but by the sustained contraction of the actin stress-fibers, which can create tension and subsequent stretching of the cell membrane [62]. Using human embryonic kidney 293T (HEK293T) cells, Wang et al. (2022) have recently demonstrated that “Piezo channels are physically linked to the actin cytoskeleton via the E-cadherin-b-catenin-vinculin mechanotransduction complex” [63]. This direct interaction between E-cadherin and mechanogating domains of Piezo1 led the authors to propose a force-from-filament model for Piezo1 activation [63]. In agreement with this tether model, earlier reports demonstrated that disruption of the actin cytoskeleton can significantly reduce Piezo1-related ion currents induced by cell indention [64].

The activity and conductivity of MA channels can also depend upon cholesterol content in the cell membrane. It has been shown that disruption of cholesterol in the membrane of myeloid K562 cells by methyl-β-cyclodextrin (MβCD) results in the decreased ion current via MA ion channels [65,66]. At the same time, reorganization of the F-actin was observed [66]. It is also interesting to note that the disintegration of the actin network using cytochalasin or latrunculin restored the ion current via MA channels in K562 cells with reduced cholesterol content in the membrane [65]. In C2C12 myoblasts, it has been demonstrated that the presence of intact lipid rafts is necessary for the normal functioning of TRPC1, while stimulation of the formation of actin stress fibers can lead to an increase in the association of TRPC1 with lipid rafts resulting in an increase in the functional stability of these channels [67].

Literature data concerning the role of cytoskeletal proteins (dystrophin, sarcoglycan, and F-actin) and cholesterol in the regulation of MA channels activity is summarized in Table 1. As shown in the table, the absence of both dystrophin and sarcoglycan appears to increase the activity of MA ion channels in myotubes. At the same time, the role of the actin cytoskeleton in the regulation of MA channels is quite ambiguous since disruption of actin with cytochalasin was shown to be associated with opposing effects on ion currents via SA channels (Table 1). As for the role of cholesterol, most of the studies showed that cholesterol depletion with MβCD results in a significant reduction in MA channel activity (Table 1).

## 4. Possible Roles of MA Channels in the Regulation of Anabolic Pathways and Protein Synthesis in Skeletal Muscles

Zanou et al. (2012) demonstrated the role of TRPC1 channels during myoblast differentiation and muscle regeneration through calcium-dependent activation of the anabolic phosphoinositide 3-kinase (PI3K)/protein kinase B (AKT)/mechanistic target of rapamycin (mTOR)/ribosomal protein S6 kinase (p70S6K) pathway during both primary myoblast differentiation and skeletal muscle regeneration after injury [71]. In addition, it was shown that under hindlimb unloading (for 14 days), a significant decrease in the content of TRPC1 and TRPC3 proteins in murine soleus muscle occurs [72,73]. It was also found that inhibition of TRPC1 expression (by siRNA injection and electroporation) deteriorated the recovery of soleus muscle mass following mechanical unloading [73]. The genetic knockout or knockdown of the TRPC1 gene in mice resulted in a decrease in the cross-sectional area of muscle fibers, as well as the content of myofibrillar proteins [71,73]. Based on the above data on the role of TRPC1 in muscle cells, it can be assumed that this molecule can participate in the conversion of mechanical forces to biochemical anabolic pathways activating muscle protein synthesis.

It is interesting to note that in response to various types of stretch (uniaxial stretch vs. multiaxial stretch) different anabolic signaling pathways can be activated in C2C12 myotubes. Troy Hornberger and co-authors showed that uniaxial stretch of the myotubes (by 15% for 10 min) leads to a significant increase in the phosphorylation levels of protein kinase B (AKT) and ERK1/2, but phosphorylation of p70 (p70S6K) (a marker of mTORC1 activity) does not change [74]. However, with the multiaxial stretch of the myotubes, a pronounced response of the mTORC1/p70S6K pathway is observed [74]. In addition, with multiaxial stretch, GSK-3β activity is suppressed (as assessed by increased inhibitory Ser9 phosphorylation) [74]. In that study, it was also found that the actin cytoskeleton might be involved in the transmission of mechanical signals (under multiaxial stretch) to p70S6K [74].

In 2006, using gadolinium and streptomycin, Spangenburg and McBride first established that functional MA ion channels (SA channels) are required for complete activation of the anabolic mTORC1/p70S6K signaling pathway in rat skeletal muscle (tibialis anterior) after a bout of eccentric contractions [75].

While investigating the effect of mechanical unloading on the transduction of mechanical signals, Tyganov et al. (2019) revealed that the anabolic response (i.e., mTORC1 activity and protein synthesis rates) of the isolated rat soleus muscle to lengthening contractions after one-day (24 h) rat hindlimb suspension is significantly diminished compared to the anabolic response of the control soleus muscle [76]. This inability of skeletal muscle to fully activate anabolic processes in response to eccentric contractions after 24 h unloading persisted with longer periods of exposure (7 days) of rats to unloading conditions [76]. Taking into account the above-mentioned work of Spangenburg and McBride (2006), it was assumed that this blunted anabolic response of unloaded skeletal muscle to mechanical stimulation could be associated with disturbances in the function of MA channels [76]. Gadolinium chloride was used in order to inhibit these channels. The treatment of the isolated soleus muscle in intact control animals with gadolinium significantly reduced the levels of p70S6K phosphorylation in response to mechanical stimuli. Interestingly, the treatment of isolated soleus muscles after a period of unloading with gadolinium did not result in a further reduction in the anabolic response to mechanical stimuli [76]. These data show that the mechanisms affected by the action of unloading are similar to the mechanisms elicited by the action of gadolinium. Consequently, the reduced magnitude of the anabolic response of the unloaded skeletal muscle to mechanical stimulation could be caused by the malfunction of the MA ion channels. It is important to note that when mechanical loading on rat hindlimbs is eliminated/reduced, a significant destruction of cholesterol rafts in the membrane of soleus muscle fibers occurs, apparently, due to the accumulation of sphingolipid ceramide [77,78]. Due to ceramide accumulation, resistance to a mechano-dependent opening of MA channels might occur. Therefore, it is possible that reduced anabolic response to a bout of eccentric contractions (“mechano-anabolic resistance”) in the unloaded skeletal muscle (the above experiment by Tyganov et al.) may be associated with the destruction of lipid rafts in the sarcolemma and subsequent decrease in the activity of MA ion channels. It has also been found that the normal function of MA ion channels is required for the full activation of anabolic signaling and the rate of protein synthesis in the postural muscle of rats during an acute recovery period following mechanical unloading [79].

The above data are summarized in Table 2. As follows from the table, MA ion channels appear to participate in the propagation of various mechanical stimuli (at least, in the form of stretching and eccentric contractions) to intracellular anabolic signaling pathways (such as the mTORC1/p70S6K pathway) and muscle protein synthesis. Future studies should provide novel information on the molecular identity of these MA channels and precise molecular mechanisms underpinning MA channels-induced regulation of muscle protein synthesis in response to mechanical stimulations of different modes.

**Figure 2:**
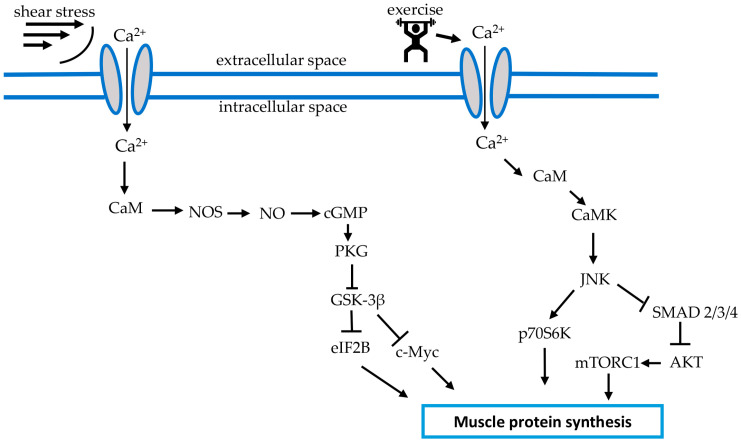
Anabolic signaling pathways proposed to be activated by MA ion channels in response to mechanical stress in the form of shear stress and exercise. Arrows indicate stimulatory signals, whereas T bars represent inhibitory signals. CaM—Ca^2+^/calmodulin, NOS—nitric oxide synthase, NO—nitric oxide, CaMK—Ca^2+^/calmodulin-dependent protein kinase, cGMP—cyclic guanosine monophosphate, PKG—protein kinase G, GSK-3β—glycogen synthase kinase 3β, eIF2B—eukaryotic initiation factor 2B, c-Myc—c-myelocytomatosis oncogene, JNK—c-Jun N-terminal kinase, SMAD 2/3/4—mothers against decapentaplegic protein, AKT—protein kinase B, mTORC1—mechanistic target of rapamycin, complex 1.

In vitro experiments on myotubes have shown that shear stress, unlike cyclic stretching by 15%, stimulates signaling processes leading to nitric oxide (NO) synthesis. At the same time, the sensing of a mechanical signal may be carried out through both integrin-related structures and MA channels [80]. Interestingly, Soltow and colleagues (2013) it was also shown that cyclic stretching of the C2C12 myotubes by 18% leads to an increase in NO production [81]. The literature describes an NO-dependent mechanism of regulation of protein and energy metabolism in skeletal muscles through the activity of glycogen synthase kinase-3 (GSK-3). An increase in NO production can inhibit the activity of GSK-3, a negative regulator of anabolic processes in the cell, via the classic NO/soluble guanylate cyclase (GC)/cyclic guanosine monophosphate (cGMP)/protein kinase G (PKG) signaling pathway [82,83,84] (Figure 2). The dependence of GSK-3β inhibitory (Ser9) phosphorylation upon NO content in the soleus muscle under conditions of reduced mechanical load (rat hindlimb unloading for 7 days) was shown by Sharlo and co-authors [85].

Of the numerous proteins studied in the mitogen-activated protein kinase (MAPK) pathway, c-jun N-terminal kinase (JNK) represents an important mechanosensitive kinase, the activity of which is directly proportional to the amount of force output during resistance exercise in human skeletal muscle [86]. Lessard et al. (2018) suggested that “JNK is a molecular switch that, when active, stimulates muscle fibers to grow, resulting in increased muscle mass” [87]. This concept is supported by data demonstrating JNK’s integration on mTOR and p70S6K signaling responses to resistance exercise in human vastus lateralis muscle [86]. One putative signaling pathway linking the exercise-induced MA channel activation and upregulation of muscle protein synthesis may include the Ca^2+^/calmodulin (CaM)/Ca^2+^/calmodulin-dependent protein kinase (CaMK)/JNK/p70S6K axis (Figure 2). Indeed, it has been shown that CaMK can activate JNK [88] and JNK, in turn, is able to activate p70S6K [89], a key kinase implicated in the regulation of mRNA translation (Figure 2). Apart from that, JNK can be involved in the regulation of protein synthesis via the JNK/SMAD2/AKT/mTORC1 pathway [87,90] (Figure 2).

## 5. Conclusions and Future Perspectives

Our understanding of the important role of MA ion channels in the process of skeletal muscle mechanotransduction and various mechanisms of their regulation via cytoskeleton and molecular modulators is a rapidly growing field of research. Yet, there remains much to be investigated in future studies. What are the precise activation mechanisms of the bona fide MA Piezo1 channels in skeletal muscle fibers? In order to obtain information about the full spectrum of Piezo1 channel function it is critical to get novel high-resolution structures that detect channels in activated and closed states. The use of genetic and chemical approaches will assist in determining the functions of specific structural domains of MA channels. Novel and highly specific activators and inhibitors (both isoform-specific and tissue-specific) of different types of MA channels are needed in order to pharmacologically target these channels for the treatment of different pathologies. At present, the use of Yoda1, a selective Piezo1 agonist, provides an intriguing avenue for further research in the field of striated muscle physiology. As MA channel activity can be changed by various chemical and physical modulators, the development of new methods of stimulation will be helpful to researchers in order to differentiate between direct and indirect modulation of various types of MA channels. In addition, there is a lack of information on how MA channel-mediated ion currents contribute to muscle cell function and dysfunction under different levels of mechanical stress (mechanical overloading vs. mechanical unloading condition). Given that skeletal muscle regeneration after injury critically depends on myogenic precursors derived from muscle satellite cells (SCs), more efforts are needed to investigate the physiological role of MA ion channels in SCs in response to various types of mechanical stressors.

To date, the contribution of MA channel activity to the regulation of such essential processes as protein synthesis, proteolysis, and regulation of the expression of the key regulatory genes in skeletal muscles remains largely unknown. Given the fundamental role of mechanical forces in the regulation of muscle protein synthesis and skeletal muscle mass, it is imperative to deepen our understanding of the possible roles of MA ion channels in the transduction of mechanical stimuli to the intracellular anabolic signaling pathways. In this regard, it appears important to investigate a putative anabolic function of MA ion channels in skeletal muscle cells. To this end, in vitro, ex vivo, and in vivo experimental models should be developed to investigate the anabolic function of MA ion channels in skeletal muscle cells/fibers. Using pharmacological and genetic instruments, it is important to establish the molecular identity of MA ion channels involved in the regulation of signaling pathways controlling muscle protein synthesis. Further, it is reasonable to investigate the effects of different modes of mechanical perturbations (fluid shear stress, cyclic strain, fiber passive-stretching, pulse electrical stimulation) on MA channel-mediated anabolic responses. In addition, it may be worthwhile to evaluate the role of cholesterol-rich lipid rafts and the actin cytoskeleton in the propagation of mechanical stimuli to anabolic signaling and protein synthesis via MA ion channels.

As for the translational potential of MA ion channels, Piezo1 and TRC channels might represent effective therapeutic targets for preventing or ameliorating skeletal muscle impairments associated with injuries, prolonged disuse/inactivity, and inherited dystrophies.

## Figures and Tables

**Table 1:** The role of cytoskeletal proteins and cholesterol in the activity of MA ion channels.

Model	Type of Exposure	Effect on MA Channels	Ref
Myotubes and isolated flexor digitorum brevis fibers	Dystrophin deficiency (mdx mice)	Channel activity in mdx myotubes is about 3–4 times greater than in wild-type myotubes. The channel open probability in mdx fibers is about 2 times greater than in wild-type fibers.	[25]
Hamster myotubes	δ-sarcoglycan deficiency	Increased activity of SA channels	[56]
HEK293 cells	Actin disruption with cytochalasin treatment	Suppressed Piezo1 activity	[64]
C2C12 myoblasts	Inhibition of actin polymerization with Rho kinase inhibitor	Decreased SA channel sensitivity	[68]
C2C12 myoblasts	Sphingosine 1-phosphate (S1P)-induced actin stress-fibers formation	Increased ion currents and conductance through SA channels	[61]
Human myeloid leukemia K562 cells	Actin disruption with cytochalasin	Decreased single currents and conductance of SA channels	[59]
Mouse ventricular myocytes	Actin disruption with cytochalasin	Decreased SA cation currents	[60]
Human gingival fibroblasts	Actin disruption with cytochalasin	Increased amplitude of stretch-activated calcium transients	[54]
Smooth muscle cells	Actin disruption with cytochalasin	Increased SA channels activity	[55]
Hamster myotubes	Actin disruption with cytochalasin	Increased SA channels activity	[56]
Murine sensory neurons	Cholesterol depletion with MβCD	Abolished slowly adapting mechanosensitive currents	[69]
Mouse myotubes	Cholesterol depletion with MβCD	Cholesterol depletion disrupted caveolae and caused an increase in MA channel current	[70]
Human myeloid leukemia K562 cells	Cholesterol depletion with MβCD	Decreased ion currents via MA ion channels	[65,66]
C2C12 myoblasts	Cholesterol depletion with MβCD	Impaired TRPC1 channel activation	[67]

**Table 2:** Effects of mechanical loading and inhibition of MA channels on the intracellular anabolic signaling in myotubes and skeletal muscles.

Model	Type of Exposure	Effect on MA Channels/Signaling Pathways	Ref
Mouse tibialis anterior muscle, primary myoblasts	Knocking out TRPC1 channels	Reduced activity of the PI3K/Akt/mTOR pathway	[71]
C2C12 myotubes	Uniaxial stretch	Increased phosphorylation of AKT and extracellular signal-regulated kinase (ERK). No change in p70S6K phosphorylation	[74]
C2C12 myotubes	Multiaxial stretch	Increased p70S6K (Thr 389) and GSK-3β (Ser 9) phosphorylation	[74]
Rat tibialis anterior muscle	Eccentric contractions and blockade of SA channels	Under SA channels blockade, decreased phosphorylation of AKT, p70S6K, and rpS6 in response to contractions	[75]
Mouse soleus muscle	Hindlimb unloading	Decreased TRPC1 protein content	[72,73]
Rat soleus muscle	Hindlimb unloading + eccentric contractions and blockade of SA channels	Blunted anabolic response (protein synthesis, mTORC1 signaling) to contractions in both unloaded muscle and muscle treated with SA channels inhibitor	[76]
Rat soleus muscle	Acute recovery from hindlimb unloading + blockade of SA channels	Reduced mTORC1/p70S6K signaling and protein synthesis rates	[79]

## Data Availability

Not applicable.
